# Influence of Multiwalled Carbon Nanotubes on the Rheological Behavior and Physical Properties of Kenaf Fiber-Reinforced Polypropylene Composites

**DOI:** 10.3390/polym12092083

**Published:** 2020-09-13

**Authors:** Farah ‘Atiqah Abdul Azam, Zakaria Razak, Mohd Khairul Fadzly Md Radzi, Norhamidi Muhamad, Che Hassan Che Haron, Abu Bakar Sulong

**Affiliations:** 1 Department of Mechanical and Manufacturing Engineering, Faculty of Engineering and Built Environment, Universiti Kebangsaan Malaysia, Bangi 43600, Selangor Darul Ehsan, Malaysia; farahatiqah@ukm.edu.my (F.‘A.A.A.); zakaria@gmi.edu.my (Z.R.); mkfadzly88@gmail.com (M.K.F.M.R.); norhamidi@ukm.edu.my (N.M.); chase@ukm.edu.my (C.H.C.H.); 2 German-Malaysian Institute, Taman Universiti Strategic Planning and Business Development, Kajang 43000, Selangor, Malaysia; 3 Malaysian Palm Oil Board (MPOB), 6 Persiaran Institusi Bandar Baru Bangi, Kajang 43600, Selangor, Malaysia

**Keywords:** rheology, fiber polymer, short kenaf fiber, kenaf, polypropylene, carbon nanotube

## Abstract

The incorporation of kenaf fiber fillers into a polymer matrix has been pronounced in the past few decades. In this study, the effect of multiwalled carbon nanotubes (MWCNTs) with a short kenaf fiber (20 mesh) with polypropylene (PP) added was investigated. The melt blending process was performed using an internal mixer to produce polymer composites with different filler contents, while the suitability of this melt composite for the injection molding process was evaluated. Thermogravimetric analysis (TGA) was carried out to investigate the thermal stability of the raw materials. Rheological analyses were conducted by varying the temperature, load factor, and filler content. The results demonstrate a non-Newtonian pseudoplastic behavior in all samples with changed kenaf fillers (10 to 40 wt %) and MWCNT contents (1 to 4 wt %), which confirm the suitability of the feedstock for the injection molding process. The addition of MWCNTs had an immense effect on the viscosity and an enormous reduction in the feedstock flow behavior. The main contribution of this work is the comprehensive observation of the rheological characteristics of newly produced short PP/kenaf composites that were altered after MWCNT additions. This study also presented an adverse effect on the composites containing MWCNTs, indicating a hydrophilic property with improved water absorption stability and the low flammability effect of PP/kenaf/MWCNT composites. This PP/kenaf/MWCNT green composite produced through the injection molding technique has great potential to be used as car components in the automotive industry.

## 1. Introduction

The kenaf plant (*Hibiscus cannabinus* L.), one of the world’s commercial crops, is presently an attractive reinforcement material in advanced composite manufacturing industries. The fibers of kenaf plants have a good mechanical strength and a durability of composites similar to many other plant types [1]. However, the low thermal decomposition temperature of natural fibers (<200 °C) is a main concern during processing. Thermal stability properties limit the suitability of polymers in the matrices of natural fibers. Polypropylene (PP) is currently the most commonly used thermoplastic polymer material in automotive component manufacturing due to its remarkable properties. A short fiber from the kenaf plant (core part) can increase the mechanical strength of PP of up to 60% [2]. Besides, our series of studies demonstrated that short kenaf fiber-reinforced composites produced by injection molding can achieve substantial physical and mechanical performance in the form of fiber-reinforced polymer composites [2,3].

Injection molding is recognized as one of the convenient and promising manufacturing processes for fiber-reinforced polymer composites. High stiffness, comparable with that of synthetic fiber-reinforced glass, was reported in previous studies [4,5]. However, before the feedstock can be injected properly, a rheological test is necessary to identify the levels of homogeneity. The flowability of the feedstock will influence moldability during the injection process. Prior study also highlighted that a small difference in fiber length (250, 450, and 850 µm) does not have much effect on the viscosity and the shorter fiber lengths ≤ 450 µm have a better interaction and distribution of fiber in the polymer matrix. Moreover, apart from the rheological studies, prior physical characterizations may reduce the constraints of thermal stability and moisture problems and enhance the mechanical and physical properties of composites. Some of the most important criteria for the fiber-polymer melts are low viscosity, good homogeneity, good fiber wetting, low moisture content, and high thermal resistance [6]. An extremely high viscosity will destruct the flowability of the feedstock during injection, whereas an extremely low viscosity may cause the formation of mold defects and residual stress, such as jetting and warping on the surface of the injected samples [7]. The residual stress mechanism can occur inside a composite material if the composites are exposed to high and unstable processing temperatures [8]. Furthermore, a high activation energy (Ea) may cause premature freezing after injection before the melt reaches the end point of the mold cavity [9].

A study showed that adding carbon nanotubes (CNTs) can improve the thermal stability of polymer composites. The prominent features of multiwalled carbon nanotubes (MWCNTs) have been widely explored by researchers. Carbon nanotubes not only contribute to the thermal property but also to the rheological, electrical, and mechanical behavior of polymers [10,11]. MWCNT fillers in low contents (<3%) can improve the flammability properties of many polymers, such as PP, ethylene vinyl acetate, polystyrene, low-density polyethylene, and polymethyl methacrylate, due to the high aspect ratios of these materials [12]. The dispersion of MWCNTs, loading rate, and mean size of nanotubes may also affect the flame retardancy properties of polymer composites. It may reduce the heat release capacity (HRC) of PP polymers [10]. HRC corresponds to material fire hazard, i.e., a low HRC means good flame retardancy.

Many of our previous studies reported the effect of kenaf filler and MWCNT addition in the enhancement of physical and mechanical properties of the composites [3,11]. The optimization of injection molding parameters was also reported in our previous studies [2,13], in which we particularly found the properties of this composite to be fascinating. Thus far, no other research has reported the rheological behavior of kenaf/MWCNT-reinforced PP composites. Therefore, the aim of this work is to demonstrate the rheological behavior of the kenaf-reinforced composite, especially the efficiency of kenaf and MWCNT as fillers. Apart from common technical factors, such as processing temperature and filler loading, a special characteristic of MWCNTs plays an important role in controlling the flow behavior of kenaf-reinforced PP composites in molten state. The water absorption and flammability properties of this composite are also determined in this study.

## 2. Materials and Methods 

### 2.1. Materials 

The PP pellet SM850 (ASTM D1238, Lotte Chemical Titan (M) Sdn. Bhd., (Kuala Lumpur, Malaysia) was used as a matrix with a melt flow index of 45 g/10 min. The kenaf fibers with a 20-mesh filler and the size of (d50) = 992.3 µm and density of 1.4g /cm^3^ was supplied by the National Kenaf and Tobacco Board (LKTN, Kota Bharu Kelantan, Malaysia). The melting temperatures of kenaf and PP were 200 and 166.82 °C, respectively. The MWCNTs with dimensions of 9.5 nm (length) and 1.5 µm (diameter) were supplied by Nanocyl SA (Sambreville, Belgium) with 90% tube purity. This MWCNT has a surface area of 250–300 m^2^/g with a volume resistivity of 10–4 Ω cm.

### 2.2. Sample Preparations 

The PP/kenaf/MWCNT composites were prepared using 10 to 40 wt % of kenaf fibers and 1 to 4 wt % of MWCNTs (Table 1) with the Sigma Blade Mixer Model Z (Winkworth Machinery Ltd., Stroudley Rd, Basingstoke, UK). The PP pellet was left melted for 15 min before adding the kenaf filler and MWCNT powder at 190 °C and mixing at 45 rpm rotation speed. The compounding process took about 30 min to ensure the homogeneity of the composites. After mixing, the melt-blended mixture (feedstock) that contains PP, kenaf, and MWCNT was allowed to cool and solidified at room temperature before it was crushed by a crusher machine into small-sized pellets. All composites were injection molded using Battenfield injection molding type BA250CDC (Awans, Belgium) using optimized parameters before the samples proceeded with the water absorption and flammability test.

### 2.3. Characterization

The thermogravimetric analyses (TGAs) were performed using the thermogravimetric analyzer Q500 (TA Instruments, New Castle, DE USA) to investigate the decomposition temperature of the raw PP, MWCNT, kenaf, and the hybrid composite. The measurements of each specimen was nearly 7.2 mg, and they were heated in nitrogen gas from room temperature to 600 °C at the heating rate of 10 °C/min. The tests were carried out in triplicate, and the results were averaged. Each TGA analysis was based on ASTM E1131 (Standard Test Method for Compositional Analysis by Thermogravimetry) [14]. The morphologies of the kenaf and hybrid composite feedstocks were examined by scanning electron microscopy (SEM, Hitachi TM-800, Hitachi, Krefeld Germany). All samples were coated with gold prior to the morphological analysis.

### 2.4. Rheological Measurements

Rheological behavior was characterized in this study to determine the flow behavior of a feedstock before the injection molding process. This property was identified using the value of flow behavior *η* and activation energy E (kJ/mol). The feedstock of the composite kenaf/PP was tested by the Shimadzu CFT 500D capillary rheometer (Hampshire, United Kingdom) with a die size of 1 mm (diameter) and a length of 10 mm In view of obtaining the low viscosity flow characteristic, the temperatures of 190–200 °C and applied loads of 20–70 N were used. All these parameters set are suggested by Lewandowski et al. [15] to analyze the pseudoplastic flow of fiber–polymer composites. The flowability index (*η*) of the kenaf/PP/MWCNT composite feedstock was calculated using Equation (1).

(1) $${\eta =k\;\gamma }^{n-1}$$

where *η* is the viscosity value, k is a constant, ү is the shear rate, and n is the fluidity index. The flow of activation energy denoted by E (kJ/mol) was determined using Equations (2) and (3);

(2) $$\eta =\eta_{0}\exp(\frac{R}{T})$$

(3) $$\ln\;\eta =\ln\;\eta_{0}+[(E/R)(1/T)]$$

where *η*_0_ is the reference viscosity, R is the gas constant (8.314 J/mol K), T is the temperature (Kelvin), and E is the flow of activation energy (kJ/mol).

### 2.5. Physical Properties of the PP/kenaf/MWCNT Composites

*The water absorption test* was conducted using the samples (dimensions of 30 mm × 30 mm × 2 mm) obtained from the feedstock of the PP/kenaf/MWCNT composite with different compositions. The specimens were first dried in an oven at 60 °C for 24 h to remove the moisture in the specimen. The initial weight of each composite specimen was recorded after the drying process. Then, the specimens were soaked in distilled water at room temperature, and the mass was measured every 24 h for 30 days. At each hour, the wet and dry surface of the specimen was weighted. Water absorption is calculated as shown in Equation (4).

(4) $$\mathrm{WA}\;(\%)=(\frac{\mathrm{Wi}-\mathrm{Wo}}{\mathrm{Wo}})\;\times \;100\%$$

where WA is water absorption, Wo is the weight of the dry specimen, and Wi is the weight of the specimen after immersion at a set time. The percentage of moisture absorption WA (%) will later be translated by plotting graphs against time changes.

The *flammability test (UL 94) with combustion test* is the first step in obtaining the plastic recognition and listing requirements in “Plastic-Recognized Components”. The UL 94 contains the 94HB, 94VB, 94–5VA, 94–5VB, and radiation panel tests [16]. The 94HB test refers to the horizontal burning methods, while the 94VB, 94VB, 94–5VA, and 94–5VB methods are used for vertical burning. The radiation panel test (ASTM E162) determines dispersion, i.e., the probability of a material to be exposed to fire [17]. The test used a 12.7 mm × 125 mm specimen held at one end in a horizontal position with markings at 25 and 125 mm from the free end. The flame was applied at the free end for 30 seconds or until the flame at the front reaches the 25 mm mark. If combustion is continuous, the time interval is set between the 25 mm mark and the 125 mm mark. If combustion stops before the 125 mm mark, the combustion time and the deformed length between the two marks was recorded. Three specimens were tested for each available composite material. The rate of combustion is calculated as shown in Equation (5).

(5) $$B=60\;\times \;\frac{D}{T}$$

where B is the burn rate (mm/min), D is the burn distance (mm), and T is the burn time (seconds).

## 3. Results and Discussion

### 3.1. Thermal Stability

**Figure 1:**
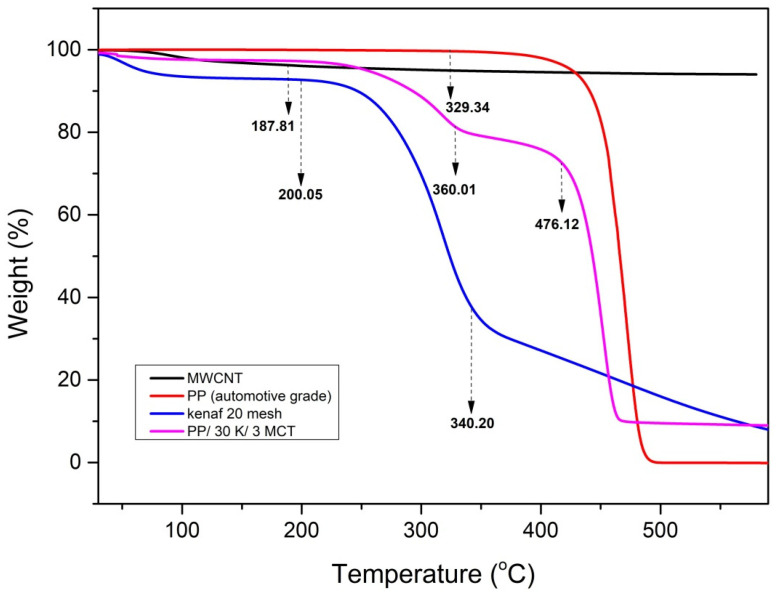
Thermogravimetric analysis (TGA) analyses of kenaf, PP, MWCNTs, and PP/kenaf/MWCNT hybrid composites.

The thermal stability of the PP, kenaf, and MWCNTs was verified using the TGA technique. As shown in Figure 1, all types of filler fibers undergo an initial decomposition between 50 and 100 °C. This early decomposition is due to the loss of moisture content in the kenaf fibers through the water vapor deposition phenomenon [18]. Subsequently, when the temperature reached 200 to 340.2 °C, all of the kenaf fillers suffered a substantial loss of approximately 60% to 70% involving the decomposition of chemical components. This result is similar to the finding of a past research, in which most natural fibers suffer from nearly 60% weight loss due to thermal decomposition in the range of 215 to 310 °C [19].

The decomposition of the PP matrix begins to occur at the temperature range of 329.34 to 529.89 °C. Meanwhile, the decomposition of the MWCNTs starts at 67.27 to 187.81 °C. It could be seen that the degradation process occurred in two steps: the composite containing 30 wt % kenaf and 3 wt % MWCNT. The initial degradation temperature at 232–345 °C is due to the degradation of cellulose and lignin, and further degradation at 360–476 °C is due to the depolymerization of PP polymer. The above results also show a notable decrease in the weight loss temperature after the addition of MWCNT fillers. The percentage of weight loss for the composite decreases about 9% compared to PP polymer. The activated MWCNT has become a barrier and radical-trapper to the free radical produced during depolymerization; thus, it delayed the decomposition of the volatile products [20]. These results concluded that the mixing and testing temperature in the rheometry should be above the melting point of PP (>166.82 °C) and at the same time lower than the decomposition temperature of the kenaf filler fibers (<200 °C).

### 3.2. Filler Loading and Load Factor Dependence of Viscosity

**Figure 2:**
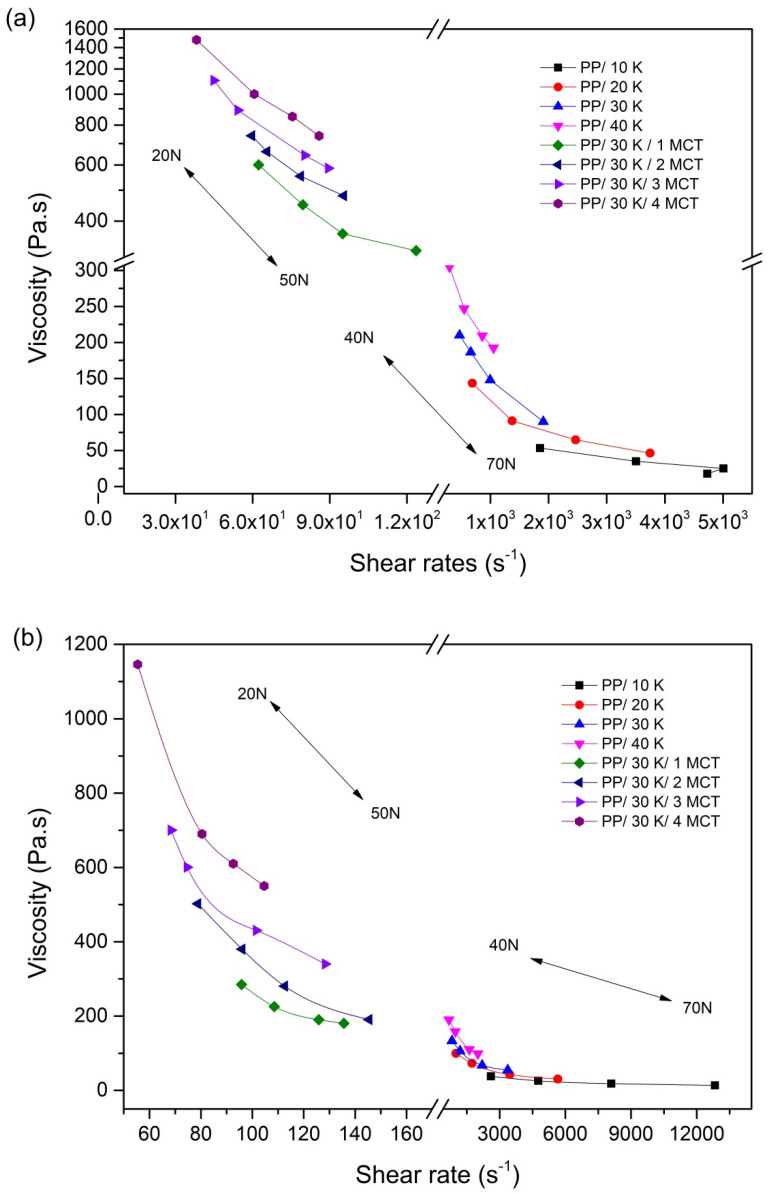
Variations in the melt viscosity of PP/kenaf and PP/kenaf/MWCNT composites at (**a**) 190 °C and (**b**) 200 °C with different shear rates.

The rheological behavior of the PP/kenaf/MWCNT feedstock toward the filler loading and load factor were studied to determine the relationship of viscosity *η* and shear rates, as depicted in the ү graph (Figure 2). In general, the PP/kenaf and PP/kenaf/MWCNT composite feedstocks are likely to produce the non-Newtonian pseudoplastic flow characteristics, as shown in Figure 2a,b. Low viscosities at 10–10^3^ Pa·s with high shear rates of approximately 102/s to 106/s correspond to the pseudoplastic flow behavior or shear thinning [7,21]. This pseudoplastic flow behavior of fiber-reinforced composites is ideal in the powder injection molding process, in which the polymeric system will be affected by the presence of fillers. Figure 2a,b show the expected dependence of viscosity for the kenaf fiber and MWCNT filler loading, in which the viscosity increases with the increase in filler loading. High-sloped melt viscosity indicates high amounts of filler, which can deteriorate the flow behavior of the feedstock.

The addition of MWCNTs has significantly altered the viscosity of the kenaf composites, especially at the low shear rates. The geometric feature of MWCNTs with the high surface area and agglomerated structure also leads to greater viscosity enhancement in the PP/kenaf composites [22]. In fact, the carbon-filled thermoplastic composites, such as non-covalent functionalized MWCNTs, usually will have better dispersion and interfacial interaction with the coupling agent maleic anhydride-grafted polypropylene (MAPP) [23]. Nevertheless, in this study, the pseudoplastic behavior of the PP/kenaf is sustained after the addition of MWCNTs. This shows that the PP/kenaf composite up to 4 wt % of MWCNTs is applicable for the injection molding process even without the addition of a coupling agent.

**Figure 3:**
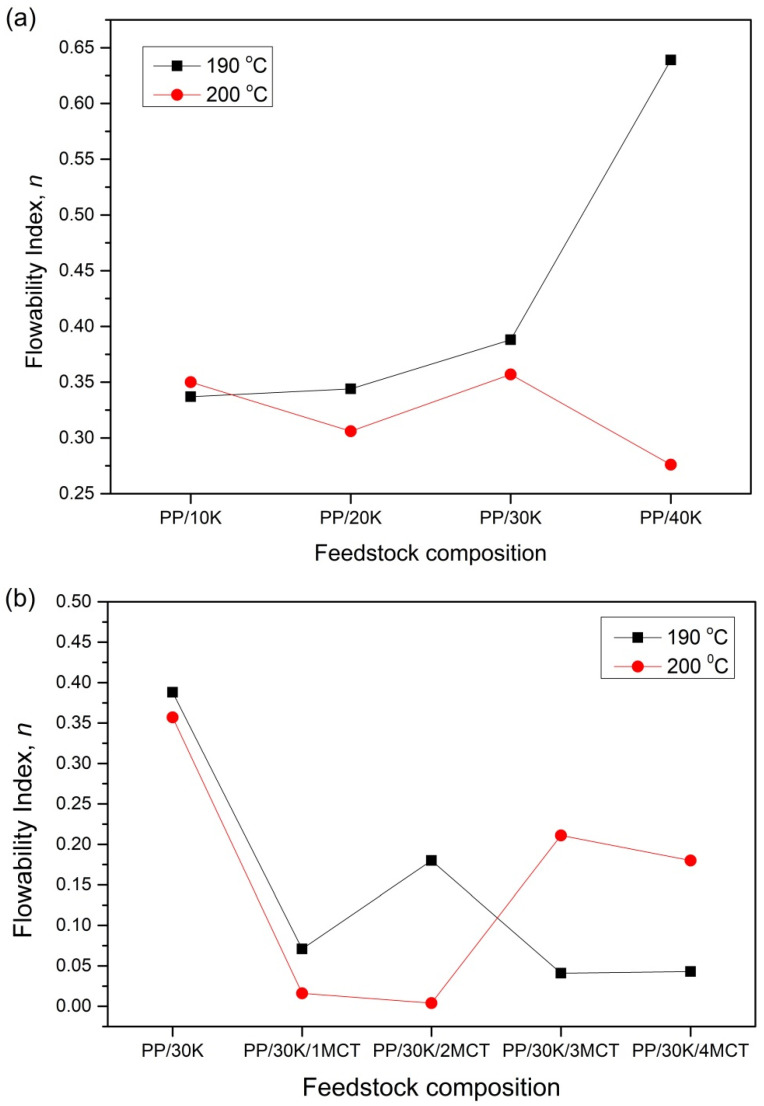
Flowability index of (**a**) PP/kenaf and (**b**) PP/kenaf/MWCNT composites at different temperatures.

The flow constraints of high filler loading can be avoided by increasing the applied load during the handling of the melt composite. Here, the applied load was able to overcome the high viscosity of the melt feedstock that was caused by the high filler loading composites. The load factor effect is less pronounced at the low filler loading, as shown by the 10 wt % of the kenaf filler in Figure 3a. This behavior can be explained on the basis of the load that acts as the shear stress, counteracting the friction or shielding the effect between the filler and matrix materials. Increasing the applied load improves the shear rates, thus reducing the viscosity of the PP/kenaf/MWCNT composite. Besides, this behavior produces good pseudoplastic flow characteristics, which can satisfy the flow characteristics of any composite feedstock [24]. Thus, this indicates that the non-Newtonian regime of kenaf-MWCNT-reinforced PP is highly dependent on the filler content and load factor.

The dependence of viscosity on temperature is irrefutable. Increasing the temperature leads to a decrease in the viscosity of the feedstock in all compositions. By raising the temperature from 190 to 200 °C, the viscosity of the PP/30 K/3 MCT composite has reduced by approximately 42%. This phenomenon can be explained by the high temperature that promotes the non-Newtonian regime through the disentanglements of molecular chains [9]. As the shear rates increase, the friction between the composite materials increases at the early stage and produces a thermal effect, which reduces the viscosity of the melt feedstock. This finding is consistent with those from other research studies on fiber-reinforced composites [25,26].

### 3.3. Reaction of Feedstock Flow Behavior Toward Temperature Changes

All the feedstock composites in this study show the values of flowability index *η* to be less than the unity values (*η* < 1), indicating the non-Newtonian pseudoplastic behavior of the composite (Figure 3a,b). A low *η* value indicates the high sensitivity of the melt composites toward the shear rates. In the presence of 40 wt % of kenaf filler at 190 °C, the flowability index further increases the resistance to flow. The result demonstrates that an excess of fiber loading content causes the reduction in the fluidity characteristic of the feedstock. The addition of an excessively high fiber load tends to form unbalanced clumps and fibers due to the weak interfacial bonding between the fiber and matrix, thus hindering the flowability of the feedstock [15]. However, as the temperature rises to 200 °C, the flowability index decreases. Our finding agrees with those of other rheological studies that state that the flow behavior of a feedstock improves when driven by the increase in shear rate, which is caused by the increase in temperature [9,27]. However, extremely high temperatures will cause a thermal decomposition of natural fibers when the process involves high processing temperatures. Thus, 30 wt % of kenaf additions was selected for further examinations on different MWCNT content in the composites.

It could be clearly seen that there was a decrease in the flowability index trend with the addition of MWCNTs. Figure 3b shows that 1 wt % of MWCNT is enough to improve the sensitivity of the PP/kenaf feedstock. The flowability index decreases from 0.388 to 0.071 at 190 °C. However, as the MWCNT filler increases to 3 wt % and the temperature increases to 200 °C, a reverse tendency is observed, leading to a higher flowability index from 0.041 to 0.211. This finding demonstrates that the excessive loading of fillers can lead to a less favorable flow of feeding material, especially at higher temperature [28]. This phenomenon is due to the non-homogeneity of composite feedstock caused by the accumulation of MWCNT filler and thermal resistance of kenaf filler. At the higher MWCNT content, the chance of attaining a more intense agglomeration is higher due to the van der Waals forces between the MWCNTs. This may develop inhomogeneity in the composite, which may affect the physical and mechanical performances of this material [20]. Thus, PP/kenaf composites with 3 wt % of MWCNT filler is favored for the injection molding process due to no difference in the flowability index at different temperatures, even as the MWCNT content increase to 4 wt %.

**Figure 4:**
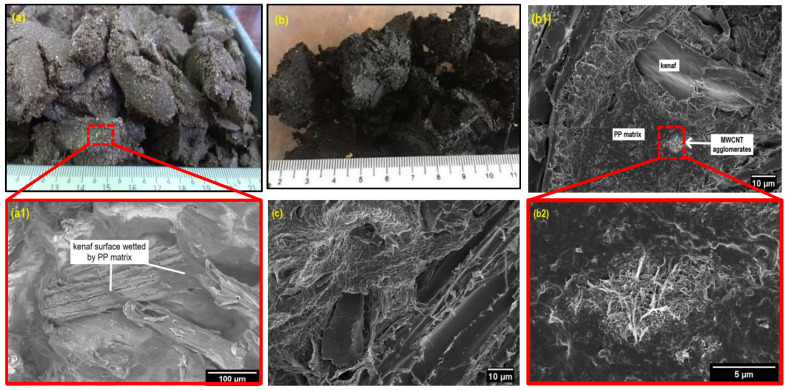
The physical state of (**a**) PP/30 K feedstock and (**b**) PP/30 K/3 MCT feedstock after mixing, with the FESEM image of (**a1**) PP/30 K and (**b1**) PP/30 K/3 MCT with the enlargement (**b2**) and the image of (**c**) PP/30 K/3 MCT with 3 wt % maleic anhydride-grafted polypropylene (MAPP) additions.

The FESEM images in Figure 4a,b show the physical state of the PP/kenaf and PP/kenaf/MWCNT feedstock after mixing. Noticeably, the surface of sample PP/30 K was perfectly wetted by the PP matrix. For PP/30 K/3 MCT, some parts were covered by the PP matrix and MWCNT, but the accumulation of MWCNT was also seen (see the bright image shown by the arrow in Figure 4b(1)). This shows that some of the MWCNT particles were not homogeneously distributed in the composite (Figure 4b). The absence of a coupling agent could be the cause of this condition. This is confirmed by the image of PP/kenaf/MWCNT with 3 wt % MAPP additions provided in Figure 4c as a reference. A homogenously distributed bright image was seen throughout the sample in Figure 4c. This is proven with the previous works reported by Razak et al. [29] whereby a better dispersion of MWCNT after adding up to a 3% MAPP coupling agent could improve the interfacial bonding between the filler and polymer of PP/kenaf-reinforced MWCNTs.

Table 2 shows that the PP/kenaf composite with a low fiber content has relatively low activation energy. As the kenaf filler content was added up to 30 wt %, the activation energy was abruptly increased to 84.853 kJ/mol. This finding indicates that the feedstock can become highly sensitive toward thermal fluctuations that may occur during the injection process. This is because the activation energy determines the moldability and sensitivity of the feedstock composites toward the temperature changes. High activation energy will cause the bad destructions in the moldability of the composites. The flow behaviors of the feedstock and activation energy certainly will affect the green part of the structure during the injection molding process. Remarkably, the addition of MWCNTs reduced by at least 35% the activation energy of the composites that contained 30 wt % of the kenaf filler. This result can reduce the sensitivity toward sudden temperature changes during processing and the chances of cracking and warping and the residual stress mechanism [30].

### 3.4. Water Absorption and Flammability Properties

**Figure 5:**
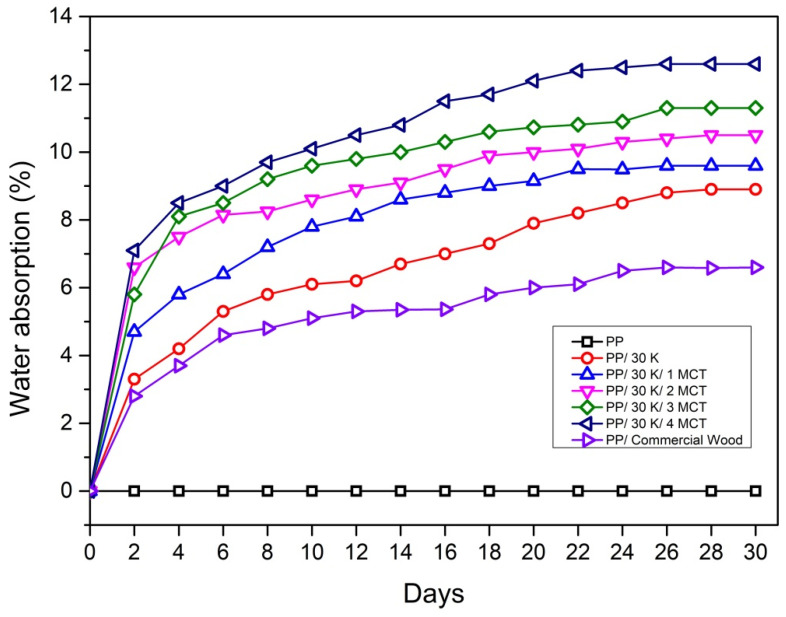
Water absorption of PP and PP/kenaf/MWCNT composites.

A water absorption test was performed to determine the water absorbance under certain conditions. The main issue in polymer–fiber composites is the compatibility between the hydrophobic polymer matrix and the hydrophilic fiber that may deteriorate composite performance. Thus, the moisture uptake behavior of the fiber composites is studied. Figure 5 shows that PP materials exhibit a negligible absorption of water because they are hydrophobic. The result showed that water absorption increases with the addition of fiber and the reduction of the polymer matrix (PP) along with the immersion time until the equilibrium is reached. The hydrophilicity of the fiber is certainly the reason for this occurrence. Surprisingly, the total amount of water absorbed by the hybrid composite also increases with the increase in MWCNT nanofiller content. The water absorption percentage rate increased for the PP/30 K filled with 1–4 wt % of MWCNTs within 4 days and continued to increase until saturation with the maximum water contents of 9.7%, 10.6%, 11.7%, and 12.6% reached after 26 days at the ambient temperature (Figure 5). The presence of MWCNTs in the kenaf composite increased the hydrophilic properties of this composite, as opposed to the PP/kenaf composite and commercial PP/wood materials.

This finding diverges from the nature of CNTs that always demonstrates hydrophobicity properties [23,31]. Several studies reported that the existence of nanofillers, such as MWCNTs, nano-SiO_2_, and nanoclay, can decrease the water absorption of the composites even without the addition of a coupling agent [32]. This situation likely happens due to the size of the MWCNT nanofiller powder that may cause aggregates, thus the strong tendency to form agglomeration. In addition, the injection molding processing technique may have needed a compatibilizer to improve the interfacial adhesion and interaction of the nanofiller, kenaf fiber, and PP matrix. Thus, in this case, some of the kenaf parts that were not fully encapsulated with MWCNTs were exposed to water, which resulted in the hydrophilic properties. This phenomenon was illustrated in a past study with a nanoclay filler in wood flour-PP composites fabricated for injection molding, which resulted in high water absorption due to inhomogeneity and fiber-matrix interaction issues [33]. Another study reported an insignificant change in water absorption in the injection of molded bagasse fiber/nano-SiO_2_ filler-reinforced HDPE when no coupling agent was added [34].

Above all, although the water absorption increases, the MWCNT addition has improved the water absorption stability as the increment in water absorption is not obvious after day 10 as compared to PP/kenaf composites. There are several factors that may influence the absorption of water such as the type of thermoplastic material, filler material, fiber content, temperature, and duration of water immersion [35]. The moisture absorption of kenaf fibers can be reduced by improving the fiber-matrix adhesion through acetylation and alkaline treatment [36]. In contrast, the addition of a coupling agent [37] or nanofillers can help improve the compatibility of the fiber and polymer matrix.

As shown in Table 3, the PP/kenaf with 3 wt % of MWCNT composite has the lowest flammability rate of 11 mm/min, as opposed to the pure PP at 18.6 mm/min. The synthetic polymers, such as PP, derived from the petroleum sources proved to have a high probability of combustion [12]. The addition of 30 wt % of kenaf fiber has increase the flammability rate, which was clearly attributed to the existence of kenaf fiber. This result indicates that MWCNT fillers can certainly reduce the flammability characteristics and hinder the high flammability of PP/kenaf composites. The presence of MWCNTs as a flame retardant has gained considerable interest among researchers. MWCNTs can be used not only as a flame retardant but also to enhance the thermal stability and mechanical properties of materials [10]. Although the dispersion of MWCNT in the PP matrix was not homogenously dispersed (Figure 4b(1)), it does not degrade the flammability properties of the hybrid composite as compared to the PP/kenaf composite. Hapuarachchi and Peijs [38] obtained comparable results, featuring a Polylactic Acid (PLA) composite reinforced by MWCNT and natural fiber with improved heat release rate and flame retardancy behavior, in which the dispersion of nanofiller was claimed to have no significant effect on the flammability properties of the polymer composites. 

## 4. Conclusions

The rheological behavior of the PP/kenaf with MWCNT composites is highly dependent on the filler content and the load factor. The changes in viscosity and flow behavior become evident with the addition of high contents (3 and 4 wt %) of MWCNT fillers. The addition of the kenaf and MWCNT filler increases the viscosity and reduces the shear rates of the melt composite, regardless of the temperature changes. However, the pseudoplastic behavior of the PP/kenaf/MWCNT composites regardless of any composition indicates the suitability of these composites for the injection molding process. Although the water absorption test has demonstrated unexpected behavior in improving the hydrophilic properties of the hybrid composites, the MWCNT addition has improves the water absorption stability of the composites. Besides, the addition of MWCNT has greatly improved the flammability characteristic of PP/kenaf up to 60%. This result demonstrated that a PP/kenaf composite with MWCNT additions has great potential to be produced using the injection molding method for automotive applications due to many aspects including affordability, lightweight components, and good thermal properties. In future investigations, the rheological behavior of a PP/kenaf/MWCNT melt composite with coupling agent additions would be interesting to be highlighted with further study on the dispersion behavior in achieving an optimum performance of PP/kenaf/MWCNT parts.

## Figures and Tables

**Table 1:** Configurations of composites. MWCNTs: multiwalled carbon nanotubes, PP: polypropylene.

Configurations	Material Compositions (%)		
	PP	Kenaf	MWCNTs
PP/10K	90	10	-
PP/20K	80	20	-
PP/30K	70	30	-
PP/40K	60	40	-
PP/30K/1MCT	69	30	1
PP/30K/2MCT	68	30	2
PP/30K/3MCT	67	30	3
PP/30K/4MCT	66	30	4

**Table 2:** Activation energy of the PP/kenaf/MWCNT composites with different compositions.

Name of Composites	Wt % of PP	Wt % of Kenaf	Wt % of MWCNT	The Activation Energy (KJ/mol)
PP/10K	90	10	-	47.558
PP/20K	80	20	-	69.712
PP/30K	70	30	-	84.853
PP/40K	60	40	-	87.405
PP/30K/1MCT	69	30	1	55.217
PP/30K/2MCT	68	30	2	33.457
PP/30K/3MCT	67	30	3	26.851
PP/40K/4MCT	66	30	4	15.687

**Table 3:** Horizontal flammability rate of pure PP and kenaf/PP composite.

Material/Composite	Length of Burnt Reach the Minimum or Maximum (mm)		Time of Burnt, t (s)	Length of Burnt, L (mm)	Flammability Rate, V (mm/min)
	25	100			
PP	Yes	Yes	242	75	18.6
PP/30K	Yes	Yes	160	75	28.1
PP/30K/3MCT	Yes	Yes	398	75	11

## References

[1] Akil H.M., Omar M.F., Mazuki A.A.M., Safiee S., Ishak Z.A.M., Bakar A.A. (2011). Kenaf fiber reinforced composites: A review. Mater. Des..

[2] Md Radzi M.K.F., Muhamad N., Sulong A.B., Haron C.H.C., Razak Z., Akhtar M.N., Ismail N.F., Tholibon D., Tharazi I. (2018). Optimization of injection molding parameters: Improving mechanical properties of kenaf reinforced polypropylene composites. J. Adv. Manuf. Technol..

[3] Razak Z., Sulong A.B., Muhamad N., Che Haron C.H., Md Radzi M.K.F., Ismail N.F., Tholibon D., Tharazi I. (2019). Effects of thermal cycling on physical and tensile properties of injection moulded kenaf/carbon nanotubes/polypropylene hybrid composites. Compos. Part B.

[4] Jin F.-L., Zhao M., Park M., Soo-Jin P. (2019). Recent Trends of Foaming in Polymer Processing: A Review. Polymers.

[5] Azaman M.D., Sapuan S.M., Sulaiman S., Zainudin E.S., Khalina A., Salit M., Jawaid M., Yusoff N., Hoque M. (2015). Processability of Wood Fibre-Filled Thermoplastic Composite Thin-Walled Parts Using Injection Moulding. Manufacturing of Natural Fibre Reinforced Polymer Composites.

[6] Väisänen T., Das O., Tomppo L. (2017). A review on new bio-based constituents for natural fiber-polymer composites. J. Clean. Prod..

[7] Nanda M., Tripathy D.K. (2012). Rheological Behavior of Chlorosulfonated Polyethylene Composites: Effect of Filler and Plasticizer. J. Appl. Polym. Sci..

[8] Chaitanya S., Singh A.P., Singh I., Lau A.K.-T., Hung A.P.-Y. (2017). Processing of lignocellulosic fiber-reinforced biodegradable composites. Natural Fiber-Reinforced Biodegradable and Bioresorbable Polymer Composites.

[9] Magalhães Da Silva S.P., Lima P.S., Oliveira J.M. (2016). Rheological behaviour of cork-polymer composites for injection moulding. Compos. Part B.

[10] Kashiwagi T., Grulke E., Hilding J., Groth K., Harris R., Butler K., Shields J., Kharchenko S., Douglas J. (2004). Thermal and flammability properties of polypropylene/carbon nanotube nanocomposites. Polymer.

[11] Razak Z., Sulong A.B., Muhammad N., Md Radzi M.K.F., Tholibon D., Tharazi I., Ismail N.F. (2017). Effect of Multi-walled Carbon Nanotube on Mechanical Properties of Kenaf/Polypropylene Composites. J. Mech. Eng..

[12] Mngomezulu M.E., John M.J., Jacobs V., Luyt A.S. (2014). Review on flammability of biofibres and biocomposites. Carbohydr. Polym..

[13] Md Radzi M.K.F., Muhamad N., Sulong A.B., Razak Z. (2017). Optimizing injection parameters of kenaf filler polypropylene composite by taguchi method. Mater. Sci. Forum.

[14] (2008). ASTM E1131-08, Standard Test Method for Compositional Analysis by Thermogravimetry.

[15] Lewandowski K., Piszczek K., Mirowski J. (2016). Rheological properties of wood polymer composites at high shear rates. Polym. Test..

[16] Tripathi D. (2002). Practical Guide To Polypropylene.

[17] (2011). ASTM E162-11, Standard Test Method for Surface Flammability of Materials Using a Radiant Heat Energy Source.

[18] Methacanon P., Weerawatsophon U., Sumransin N., Prahsarn C., Bergado D.T. (2010). Properties and potential application of the selected natural fibers as limited life geotextiles. Carbohydr. Polym..

[19] Suardana N.P.G., Ku M.S., Lim J.K. (2011). Effects of diammonium phosphate on the flammability and mechanical properties of bio-composites. Mater. Des..

[20] Yaghoobi H., Fereidoon A. (2019). Preparation and characterization of short kenaf fiber-based biocomposites reinforced with multi-walled carbon nanotubes. Compos. Part B.

[21] Kiziltas A., Company F.M., Han Y., Gardner D.J. The Effect of Filler Type on the Mechanical, Thermal and Rheological Properties of Cellulose-filled Thermoplastic Composites. Proceedings of the International Convention of Society of Wood Science and Technology and United Nations Economic Commission for Europe-Timber Committee.

[22] Choi J.H., Jegal J., Kim W.N. (2006). Fabrication and characterization of multi-walled carbon nanotubes/polymer blend membranes. J. Memb. Sci..

[23] Xie X.L., Mai Y.W., Zhou X.P. (2005). Dispersion and alignment of carbon nanotubes in polymer matrix: A review. Mater. Sci. Eng. R Rep..

[24] Ramli M.I., Sulong A.B., Muhamad N., Muchtar A., Zakaria M.Y. (2017). Rheological Properties of Titanium Alloy (Ti6Al4V)-Wollastonite (W) Composite Using Palm Stearin as Based Binder. J. Mech. Eng..

[25] Abdennadher A., Vincent M., Budtova T. (2016). Rheological properties of molten flax- and Tencel^®^-polypropylene composites: Influence of fiber morphology and concentration. J. Rheol..

[26] El-Sabbagh A., Ramzy A., Steuernagel L., Meiners D. (2016). Flowability and fiber content homogeneity of natural fiber polypropylene composites in injection molding. AIP Conf. Proc..

[27] Sojoudiasli H., Heuzey M.C., Carreau P.J. (2014). Rheological, morphological and mechanical properties of flax fiber polypropylene composites: Influence of compatibilizers. Cellulose.

[28] Islam M.A., Begum K. (2015). Rheological Behavior of Coir-Fiber-Filled Polypropylene Composites at Constant Shear Stress. Polym. Compos..

[29] Razak Z., Sulong A.B., Muhammad N., Che Haron C.H., Md Radzi M.K.F., Tholibon D., Tharazi I., Ismail N.F. (2018). The Effects of Maleic Anhydride Grafted PP (MAPP) on the Mechanical Properties of Injection Moulded Kenaf/CNTs/PP Composites. Sains Malays..

[30] Azaman M.D., Sapuan S.M., Sulaiman S., Zainudin E.S., Abdan K. (2013). An investigation of the processability of natural fibre reinforced polymer composites on shallow and flat thin-walled parts by injection moulding process. Mater. Des..

[31] Clark M.D., Krishnamoorti R. (2012). Near-superhydrophobic behavior of multi-walled carbon nanotube thin films. Thin Solid Films.

[32] Devnani G.L., Sinha S. (2019). Effect of nanofillers on the properties of natural fiber reinforced polymer composites. Mater. Today Proc..

[33] Ashori A., Nourbakhsh A. (2011). Preparation and characterization of polypropylene/wood flour/nanoclay composites. Eur. J. Wood Prod..

[34] Hosseini S.B., Hedjazi S., Jamalirad L., Sukhtesaraie A. (2014). Effect of nano-SiO_2_ on physical and mechanical properties of fiber reinforced composites (FRCs). J. Indian Acad. Wood Sci..

[35] Maslinda A.B., Abdul Majid M.S., Ridzuan M.J.M., Afendi M., Gibson A.G. (2017). Effect of water absorption on the mechanical properties of hybrid interwoven cellulosic-cellulosic fibre reinforced epoxy composites. Compos. Struct..

[36] Tholibon D., Tharazi I., Sulong A.B., Muhamad N., Ismial N.F., Radzi M.K.F.M., Radzuan N.A.M., Hui D. (2019). Kenaf Fiber Composites: A Review on Synthetic and Biodegradable Polymer Matrix. J. Kejuruter..

[37] Alam M.S., Roy S.K., Khan G.M.A., Haque M.A., Haque M.I., Gafur M.A. (2017). Effect of Chemical Treatments and Coupling Agents on the Properties of Unidirectional Jute Fiber Reinforced Polypropylene Composite. J. Kejuruter..

[38] Hapuarachchi T.D., Peijs T. (2010). Multiwalled carbon nanotubes and sepiolite nanoclays as flame retardants for polylactide and its natural fibre reinforced composites. Compos. Part A.

